# Genipin improves reproductive health problems caused by circadian disruption in male mice

**DOI:** 10.1186/s12958-020-00679-9

**Published:** 2020-12-11

**Authors:** Yihua Xu, Li Wang, Siyuan Cao, Ruihua Hu, Rui Liu, Ke Hua, Zhigang Guo, Hong-Jie Di, Zhigang Hu

**Affiliations:** 1 Jiangsu Key Laboratory for Molecular and Medical Biotechnology, College of Life Sciences, Nanjing Normal University, 1 WenYuan Road, 210023 Nanjing, China; 2 Department of Endocrinology, The Second Affiliated Hospital of Nanjing University of Traditional Chinese Medicine, 210017 Nanjing, China

**Keywords:** Circadian disruption, Fertility, Spermatogenesis, Male mice, Genipin

## Abstract

**Background:**

Circadian rhythm disruption impacts a wide range of physiological processes, including fertility. However, the effect of circadian disruption on male spermatogenesis and fertility, and treatments for these effects have been largely unexplored at the molecular level.

**Methods:**

In this study, we examined the effects of genipin on improving the reproductive health problems caused by circadian disruption. Three groups of animals were fed under different conditions: control group (normal T cycle with saline), group of shortened T cycles (Light/Dark = 4 hours/4 hours) with saline, and a group of shortened T cycles with genipin by oral gavage. The male fertility was evaluated by fertility study and pups parameters analysis after successful sexual behavior and mating with female mice. We sacrificed the treated animals after 5 or 10 weeks and collected the testis, sperm and serum for histological analysis, sperm motility assay, and serum hormone detection, respectively. Furthermore, the effect of genipin was assessed by detection of progesterone secretion and steroidogenic key proteins expression, including StAR and CYP11A1, in mouse Leydig tumor MLTC-1 cells.

**Results:**

Male mice exposed to shortened light-dark cycles, much shorter than 24 hours, had reduced fertility with decreased sperm concentrations and sperm motility. Male mice under circadian disruption have reduced testis size and abnormal morphology, leading to lower fertility rates, reduced litter size and pup body weight. Treatment with exogenous genipin, a natural plant-derived compound, alleviated circadian disruption-induced damage to fertility and spermatogenesis and normalized testosterone, dihydrotestosterone (DHT), and androstenedione (ASD) levels in the male mice. The levels of key proteins involved in steroidogenesis, StAR and CYP11A1, were reduced in mouse testes after the circadian disruption, but genipin treatment restored the reduction. The mRNA expression of SRD5A1, which encodes an androgen synthesis enzyme, was also upregulated by genipin treatment. Furthermore, genipin treatment showed a positive effect on steroidogenesis in MLTC-1 cells, resulting in an increase in hormone secretion and the upregulation of StAR and CYP11A1.

**Conclusions:**

Our results showed an association between circadian disruption and reproductive health problems in male mice and indicated that treatments with genipin have positive effects on the reproductive health of male mice with circadian rhythm disorders.

## Introduction

Circadian rhythms are a fundamental feature in eukaryotes and coordinate numerous aspects of behavior, physiology, and metabolism [1]. The circadian clock system occurs at all levels of cellular organization, allows an organism to interact with external stimuli on the cell, organ, and organism levels, according to a transcriptional-translational feedback loop by light-dark cycles [2]. Physiologically, these 24-h circadian rhythms are orchestrated by a primary clock in the suprachiasmatic nucleus (SCN) of the hypothalamus in mammals. Circadian clocks comprises of a set of proteins such as Bmal1, Per1, Clock, Cry1 and Cry2, by which one or more gets acutely sensitive to light, resulting in an oscillator that can synchronize over local time [1, 2]. The circadian clock allows organisms to anticipate the predictable changes in the environment that occur at approximately the same time of day or adapt to daily variations in their environment. This intricate network enables the adjustment of principal physiological pathways so that an appropriate physiological or behavioral response can induce at the correct time. Several kinds of disorders such as diabetes, bipolar disorder, attention deficit hyperactivity disorder (ADHD), and inflammatory bowel disease, reports being associated with disturbances in the circadian rhythms [3–6].

Circadian rhythm disruption affects a wide range of physiological systems in mammals. Reproductive physiology, in particular, is profoundly under the influence of circadian rhythms [7, 8]. Circadian disruption is a risk factor for impaired reproduction and fertility in both men and women. Environmental circadian disruption (ECD) has been recognized as a risk factor for fertility problems among working women, and chronic ECD is associated with abnormal reproductive cycles, irregular menstrual cycles, increased risk of endometriosis, increased latency to pregnancy, miscarriages, low birth weight or preterm delivery, and a reduced incidence of breastfeeding [9, 10]. The fertility of males has declined over the past four decades. Although half of the cases of male infertility remain unexplained, emerging evidence implicates sleep disturbance in the risk of male infertility [11, 12]. Indeed, the prevalence of circadian disturbance appears to be increasing in parallel with a decline in sperm quality, a commonly used surrogate marker of male fertility [12]. Furthermore, a case-control study indicated that genetic variability in the *Clock* gene associates with male infertility, implying the role of the circadian timing system in human reproduction [13]. Clock gene expression occurs in tissues of the hypothalamic-pituitary-gonadal (HPG) axis. Knockdown of *Clock* gene expression in the testes of male mice led to small litter size, low in vitro fertility rate, low blastula formation rate, and low acrosin activity of sperm with *Clock* knockdown [14]. Male *Bmal1* KO mice show infertility, with smaller testes and reduced sperm counts than normal males [7].

In mammals, circadian rhythms are involved in the regulation of serum concentrations of many reproductive hormones, which are critical in mammalian fertility [15, 16]. Steroidogenesis or biosynthesis of steroid hormones is a complex multistep process by which precursor cholesterol is converted to pregnenolone and subsequently metabolized into other biologically active steroids in steroidogenic tissues, including adrenal and gonads [17]. The limited step of steroidogenesis is cholesterol transfer from outer mitochondrial membrane (OMM) to the inner mitochondrial membrane (IMM), which is dependent upon the steroidogenic acute regulatory protein (StAR) [18]. Several other enzymes or proteins are involved in the process of steroidogenesis, such as cytochrome P450 side-chain cleavage enzyme (CYP11A1, carries out the first committed step in steroidogenesis, i.e., conversion of cholesterol to pregnenolone), steroid 5 alpha-reductase 1 (SRD5A1, converts testosterone to dihydrotestosterone) and hydroxysteroid 17-beta dehydrogenase 1 (HSD17B1, catalyzes ASD to testosterone) [17]. The levels of several hormones fluctuate according to the light and dark cycle and are also affected by sleep [19]. Shift work modified the luteinizing hormone (LH) surge and follicle-stimulating hormone (FSH) levels, which might be related to the misalignment of cortisol and melatonin rhythms during shift work [20, 21]. The dysregulation of reproductive hormone levels by circadian disruption contributes to low fertility or infertility [21]. While the relationship between circadian rhythms and female pregnancy being well studied [8, 22, 23], the effect of circadian disruption on spermatogenesis and fertility in males and treatments that addresses circadian disruption on fertility needs exploration.

Genipin, a natural aglycon of geniposide and an active constituent of *Gardenia jasminoides Ellis*, has multipurpose biological and pharmacological activities, such as antioxidant, antimicrobial, anti-inflammation, antitumor, neuroprotective and hepatoprotective effects [24–29]. Additionally, genipin has been widely used as a natural crosslinker for tissue engineering [30]. Current studies implicated genipin in the regulation of steroid hormone secretion and lipid metabolism [27, 31]. Moreover, genipin inhibited the expression of mitochondrial uncoupling protein 2, which affected androgen synthesis and was associated with infertility [31, 32]. These reports implicated genipin in the regulation of reproduction and fertility in mammals. Here, we studied the effect of circadian disruption on spermatogenesis and fertility in male mice. We showed that circadian disruption impaired spermatogenesis and fertility in male mice, and genipin ameliorated sperm motility and fertility under circadian disruption conditions.

## Materials and methods

### Animal and housing

Eight to ten-week-old male and female ICR mice were obtained from the Model Animal Research Center of Nanjing University (Nanjing, Jiangsu, China). Mice were initially housed under a 12-h light/dark cycle (12:12 LD) at 25 °C with normal laboratory diet. The light-dark (LD) cycle was set as described previously, with minor modifications [33]. Briefly, male mice were randomly divided into three groups after a minimum 1-week acclimation period: normal T cycles (T = 24 hours, L:D = 12 hours:12 hours) with saline (normal control, NC), shortened T cycles (T = 8 hours, L:D = 4 hours:4 hours) with saline (disrupted + saline, SC), and shortened T cycles with GNP (25 mg·kg^− 1^) (disrupted + GNP, SG). GNP (C_11_H_14_O_5_, HPLC ≥ 98%; Zelang, Nanjing, Jiangsu, China) was dissolved in saline solution and given to mice at the end of every light cycle by oral gavage until the mice were used for the sperm quality assay [27]. Equal volumes of saline solution were infused into mice as a control. Mouse body weights and food intake were monitored every two days.

### Cell culture and treatment

To study the function of GNP on steroidogenesis, the mouse leydig tumor MLTC-1 cells were cultured and treated with GNP. MLTC-1 cells were obtained from the American Type Culture Collection (ATCC, Manassas, VA). MLTC-1 cells were cultured in RPMI-1640 media supplemented with 10% fetal bovine serum (FBS), and 100 unit/ml penicillin and 100 µg/ml streptomycin. Cell cultures were maintained at 37 °C in a humidified incubator in the presence of 5% CO_2_/95% air. For treatment, cells were plated and cultured in 6-well or 12-well plates with GNP (20 µM, dissolver in DMSO) for varying incubation time. The supernatants of cultured media were collected for progesterone measurement, and cells were collected for RNA and protein extraction.

### Fertility study

For male fertility evaluations, male mice were subjected to different T cycles and treatments for 9 weeks, and each male mouse was kept with two virgin females together. The female mice were obtained from the Model Animal Research Center of Nanjing University and were randomly divided into three groups for mating after a minimum 1-week acclimation period. Mating behavior was observed in the Plexiglas cage with a dim red light source. Mating behavior was observed under a normal T cycles to avoid a short-term effect of circadian disturbance on female mice. Additional female mice were used if the original female mice were unreceptive to mating. After approximately a whole LD cycle with successful sexual behavior and mating confirmed by the presence of vaginal plugs or the presence of sperm in vaginal smears, the males were separated from the females, and the females were maintained until spontaneous delivery. The day of delivery was considered gestational day 0. The numbers of pups were counted, and pup sex, body weight and body length were measured. The fertility index was calculated by the following equation [34]: fertility index = number of females that gave birth/number of copulated females × 100%.

### Sperm parameters

The males were fed for five or ten weeks under normal T cycles or shortened T cycles with the administration of GNP or saline. Then, the animals were sacrificed by cervical-dislocation. Both sides of the epididymides cauda were quickly removed and immediately crumbed by syringe needles within the 0.2 ml of physiological saline into a petri dish to release spermatozoa. Sperm concentration and sperm motility were assessed by computer-assisted semen analysis (CASA, WLJY-9000, Weili New Century Technology Development Co., Ltd., Beijing China). A Macro sperm-counting chamber (Nanjing Yuancheng Company, Nanjing, China) was used to load the spermatozoa samples. Average path velocity (VAP), curvilinear velocity (VCL), straight-line velocity (VSL), and beating cross frequency (BCF) were calculated for the sperm of each group of mice by analyzing three recordings of at least 100 spermatozoa.

### Histological analysis

The testicular tissues were collected and fixed in 4% paraformaldehyde solution. Paraffin-embedded samples were cut into 5 µm transverse sections for routine hematoxylin & eosin (H&E) staining. All the stained sections were examined by light microscopy (× 200 magnification).

### Reproductive hormones

The cell culture media were centrifuged at the speed of 5000r.p.m for 5 min and supernatant were collected for progesterone measurement using the Pg ELISA kit (E-EL-0090c, Elabscience Biotechnology Co.,Ltd, Wuhan, China). Blood samples were collected from the right retroorbital plexus of different treated mice and centrifuged at the speed of 3000r.p.m for 10 min at 4℃. The serum levels of testosterone (#SBJ-M0439-96T), dihydrotestosterone (DHT) (#SBJ-M0909-96T), androstenedione (ASD) (#SBJ-M0437-96T), and gonadotropin-releasing hormone (GnRH) (#SBJ-M0558) were determined using commercial kits (SenBeiJia Biological Technology Co., Nanjing, China) according to manufacturer’s instructions. The serum melatonin levels were tested by a commercial ELISA kit from Shanghai Haling Biological Technology Co. LTD (Shanghai, China).

### Real-time quantitative PCR

Total RNA was extracted from testicular tissues or MLTC cells using Trizol reagent (Invitrogen, USA). Total RNA (1 µg) samples were reverse-transcribed using superscript II reverse transcriptase (Life Technologies, Grand Island, NY). Amplification of cDNAs was performed using a qPCR kit (TAKARA Biotechnology, catalog no. RR420A) and gene-specific primers on an ABI StepOneplus system according to manufacturer’s instructions [35]. 36B4 was used as an internal control. All primer sequences used for qPCR are presented in Table 1.

**Table 1:** Primers for qPCR

Name	Sequence
StAR	5’-CGGAGCAGAGTGGTGTCATC-3’-F 5’-TGAGTTTAGTCTTGGAGGGACTTC-3’-R
CYP11A1	5’-ACTGTGAACTGAAGGCTGG-3’-F 5’-GGGAAAGAGGGAAAGAGGATG-3’-R
SRD5A1	5’-ACTTGCAGAGCCGATACTTG-3’-F 5’-TTTCTCAGATTCCGCAGGATG-3’-R
HSD17B1	5’-GGAATTGGATGTCAGAGACTCC-3’-F 5’-CCCACAGCGTTCAATTCATG-3’-R
36B4	5’-TTTGGGCATCACCACGAAAA-3’-F 5’-GGACACCCTCCAGAAAGCGA-3’-R

### Western blot analysis

Testicular tissues were collected and lysed in RIPA buffer using GeneReady Ultracool system (Life Real, Hangzhou, China). Equal amounts of protein (10 ~ 15 µg) were loaded on a 12% SDS-polyacrylamide gel and blotted onto PVDF membranes. The membranes were blocked with 5% skim milk in phosphate buffer saline (PBS) with 0.1% Tween-20 for 1.5 h and incubated with anti-StAR antibody (1:400) (A1035, ABclonal, Wuhan, China), anti-CYP11A1 antibody (1:200) (13363-1-AP, Proteintech, Wuhan, China) or anti-vinculin antibody (1:1000) (Abways Technology, Inc., Shanghai, China) overnight at 4℃. Then the membranes were washed with PBS for three times and probed with HRP Goat Anti-Rabbit lgG (1:5000) (ABclonal) for 1.5 h. The blots were developed via Tanon-4500 luminescent imaging workstation (Tanon Science & Technology, Shanghai, China).

### Statistical analysis

Statistical analysis was performed with GraphPad Prism 6.0. Data were analyzed for normality using the Kolmogorov-Smirnov test, and homogeneity of variances was analyzed by Bartlett’s test [35, 36]. Significant differences were identified using Student’s *t*-test or analysis of variance (ANOVA), in the case of comparisons among more than two groups. Statistical analyses were performed using ANOVA followed by Bonferroni’s post-test. A *p* value of < 0.05 was considered as statistically significant. All data are expressed as the mean ± SD [35].

## Results

### Induction of an animal circadian disruption model by a shortened T cycle

**Fig. 1:**
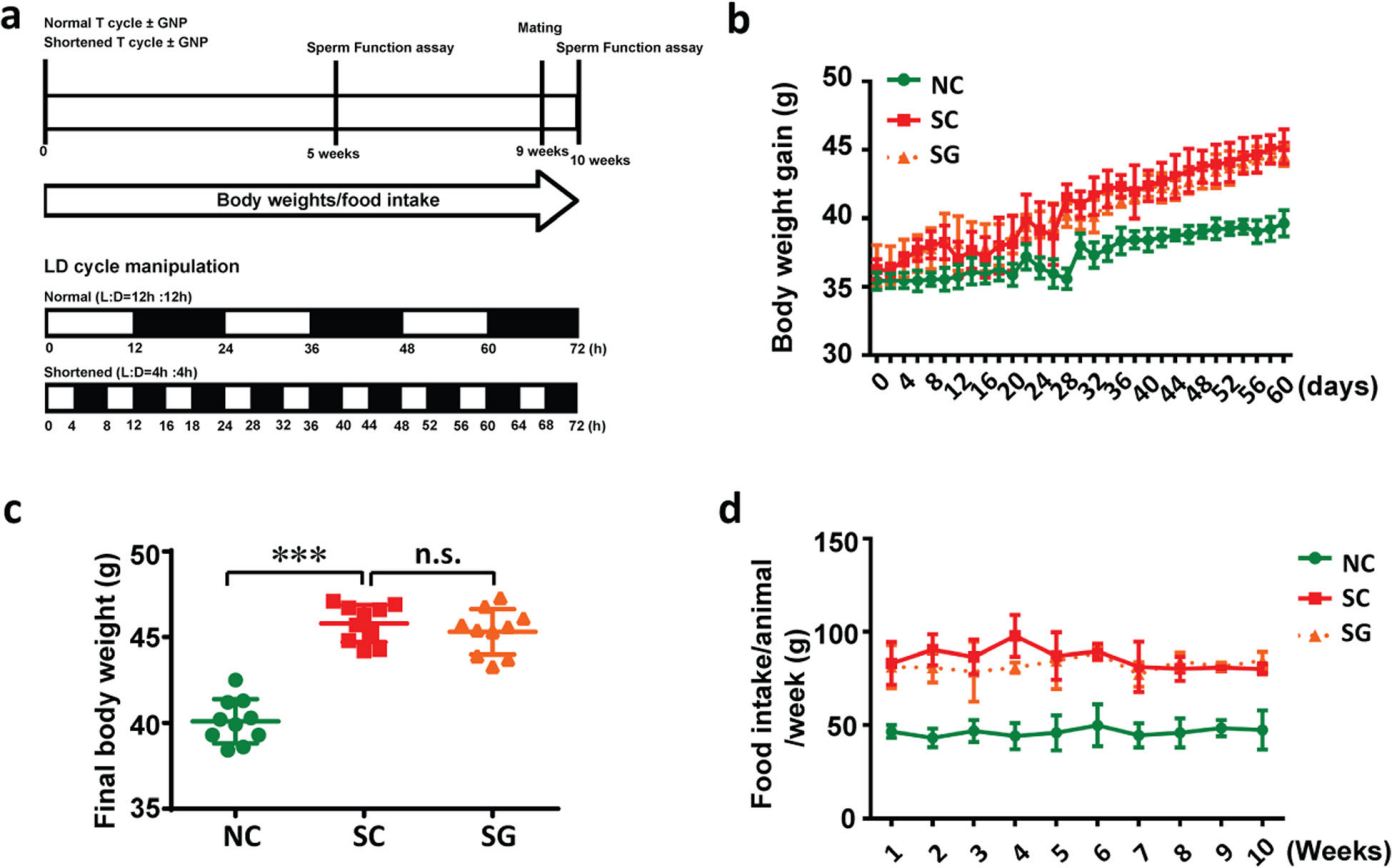
Experimental scheme for different LD cycle manipulations, treatments and monitoring of mice. **a** Scheme of experiment design. (b-d) Effects of circadian disturbance and genipin on (**b**) the weight gain, (**c**) body weight, and (**d**) food intake of mouse model. ***P < 0.001

To assess the impact of circadian disruption on fertility, we subjected male ICR mice to normal/shortened T cycles for ten weeks, as described in the Method section (Fig. 1a). We measured daily food consumption and body weight every two days. Figure 1b and c show that the average final body weight and weight gain were significantly higher in the mice subjected to shortened T cycles (L:D = 4 hours:4 hours) than those subjected to normal T cycles (L:D = 12 hours:12 hours). However, there was no difference in the final body weight and body weight gain of mice between the SG group and SC group. Similarly, both SC mice and SG mice showed higher daily food consumption than NC mice (Fig. 1d).

### Fertility parameters

To analyze the fertility of male mice subjected to shortened T cycles and GNP treatment, we compared the fertility rates of NC, SC, and SG male mice through natural mating. Only 50% (*n* = 20) of female mice that mated with SC males produced offspring, whereas 75% (*n* = 20) of females that mated with SG males and 95% (*n* = 20) of females that mated with NC males produced offspring. These data indicated a significant decrease in the fertility of mice subjected to shortened T cycles, and genipin relatively fixed the circadian disruption-impaired fertility of male mice. Moreover, the number of pups per litter of females that mated with SC mice was lower than that of those mated with NC mice (9.4 ± 0.92 vs.12 ± 1.12, *n* = 20, *P* < 0.001) (Table 2). The litter size of females that mated with SG mice was rebuilt (12.67 ± 1.14). Additionally, the average body weight of the pups of SC mice was markedly lower than that of NC mice pups (1.57 ± 0.23 g vs. 1.79 ± 0.16 g, *P* < 0.001), whereas genipin treatment regained pup body weight.

**Table 2:** Fertility parameters. All data are expressed as mean±SD. ^a^ indicates a statistical difference when compared with the NC group (*P*<0.001), ^b^ indicates a statistical difference when compared with SC group (*P*<0.001)

Fertility parameters	NC (Control)	SC (Disrupted + saline)	SG (Disrupted + GNP)
No. of housed male	10	10	10
No. of housed female	20	20	20
Fertility index (%)	95 (19/20)	50 (10/20)^a^	75 (15/20)^b^
Litter size	12 ± 1.12	9.4 ± 0.92^a^	12.67 ± 1.14^b^
Pups body weight (g)	1.79 ± 0.16	1.57 ± 0.23^a^	1.803 ± 0.223^b^
Pups body length (cm)	4.31 ± 0.63	4.42 ±0.22	4.59 ± 0.19^b^

### Changes in the gross anatomy of the testes

**Fig. 2:**
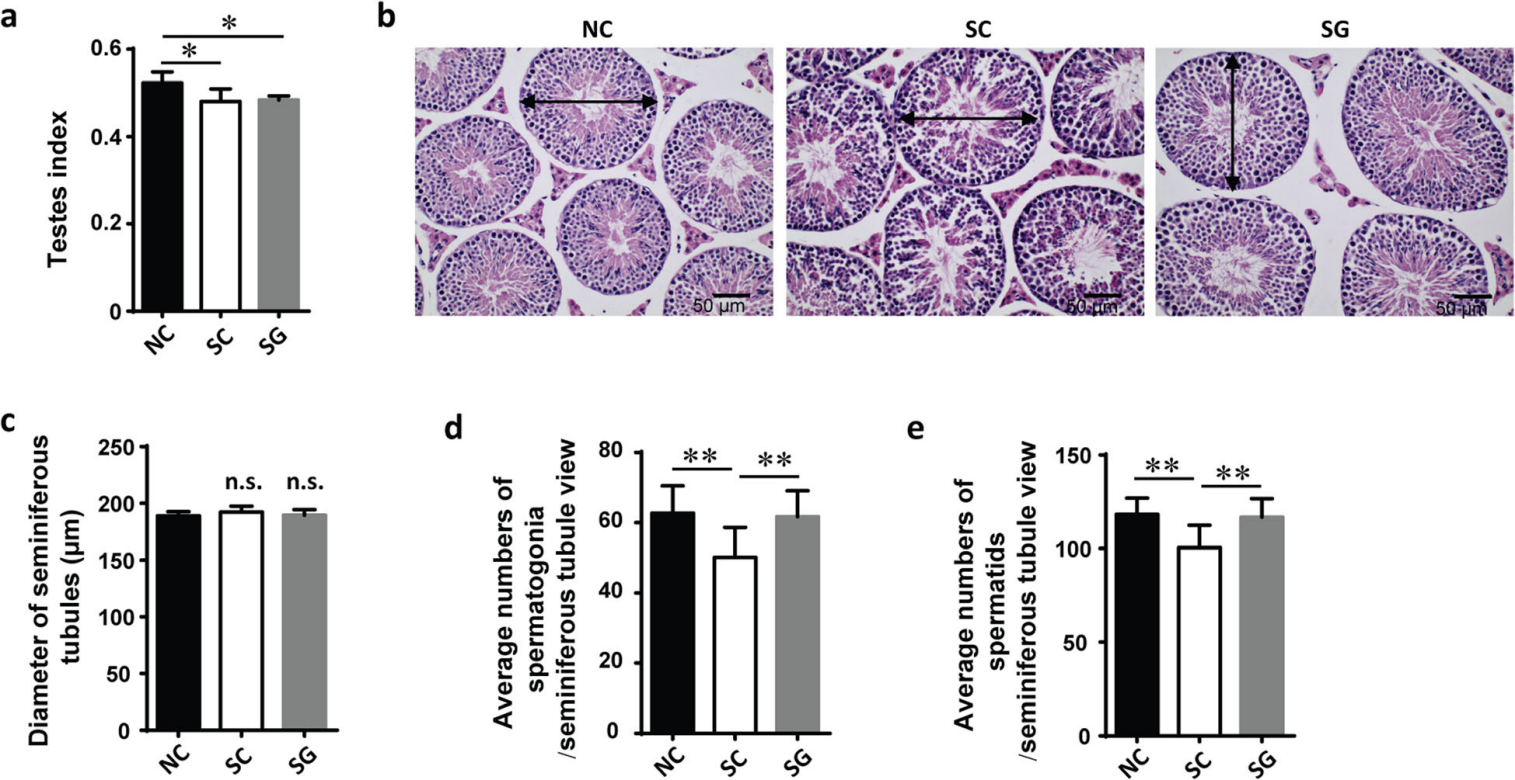
Morphometric analysis of testes. **a** Testes index of three groups. (Testis index = Testis weight/Body weight × 100). **b** Histology cross-sections of seminiferous tubules. The seminiferous tubules diameters were indicated by double-headed arrows. **c** Average diameter of seminiferous tubules. Average number of spermatogonia cells (**d**) and spermatid cells (**e**) per section (*n* = 5 per group; about 30 seminiferous tubules were examined for each mouse). *P < 0.05; **P < 0.01

After ten weeks of treatment, the average body weight of male SC mice was increased compared with that of NC mice, while the average weight of the testes was slightly decreased (Fig. 2a). Histological examination of the testes showed that the diameter of seminiferous tubules remained similar among the three groups (Fig. 2b and c). We observed six to eight layers of aligned spermatogenetic cells in the seminiferous tubules. The characteristic of leptotene, zygotene, pachytene, diplotene, and diakinesis was observed in the H&E staining of cross-sections of seminiferous tubules. We found marked atrophy of the seminiferous tubules and more vacuoles in the SC group than in the NC group, whereas genipin treatment alleviated this damage (Fig. 2b). The average numbers of spermatogonia and spermatids were reduced significantly in male SC mice (*p* < 0.05) compared with male NC mice, and genipin treatment reestablished these effects on these cells (Fig. 2d and e).

### Semen analysis

**Fig. 3:**
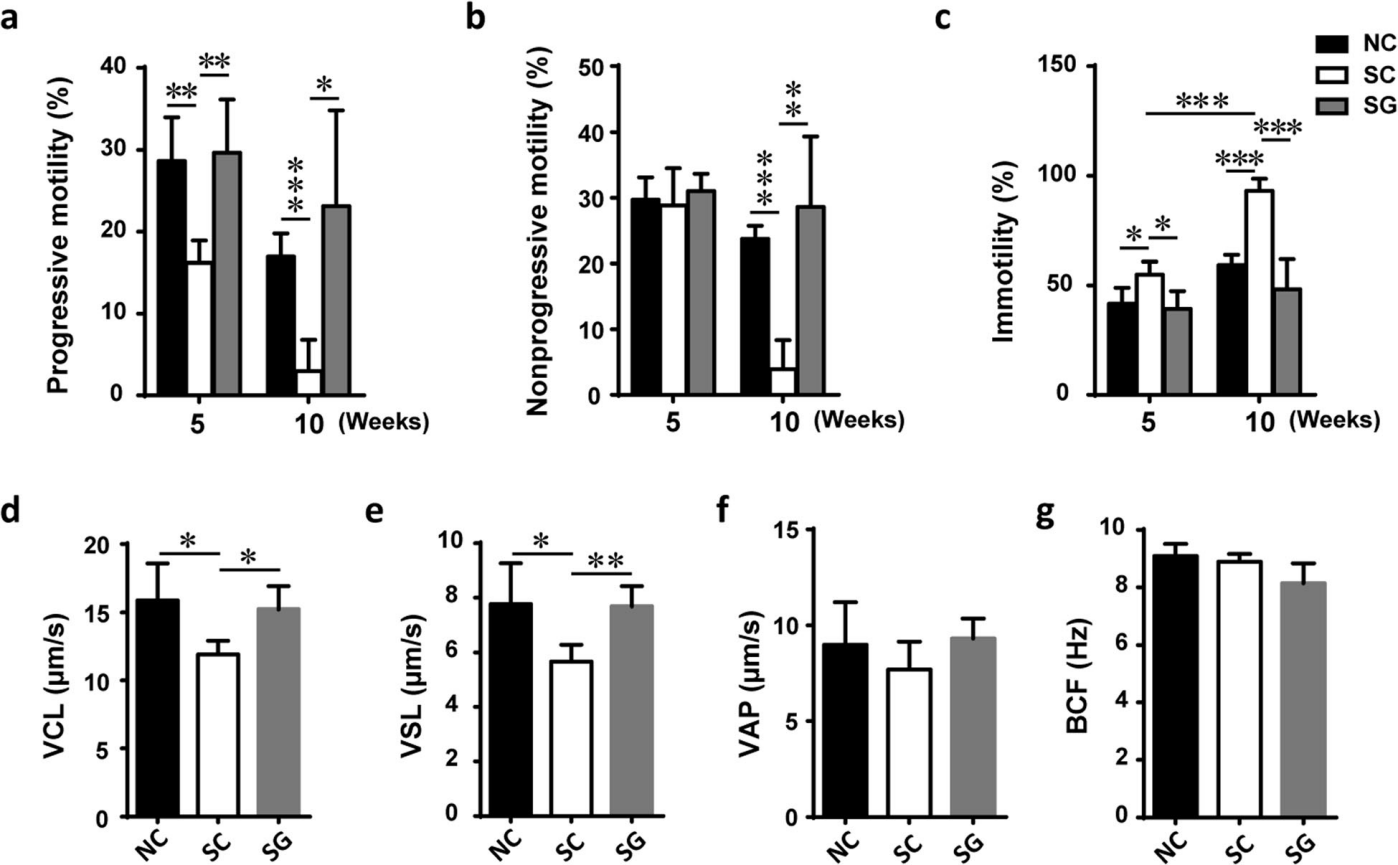
Sperm parameters. **a** Progressive motility, **b** non-progressive motility (**c**) immotility of sperms from control and treated mice for five or ten weeks. **d-g** The VCL, VSL, VAP, and BCF of sperms from control or treated mice for five weeks. *P < 0.05; **P < 0.01; ***P < 0.001

Finally, to evaluate whether the damaged testes subsequently affected sperm generation and function, we isolated sperm and analyzed the concentration, motility and motion parameters of the sperm by CASA. Compared with that of male NC mice, the average sperm concentration of male SC mice significantly reduced to 33% (*P* < 0.001), and genipin reversed this effect (Table 3). Moreover, sperm motility was impaired by circadian disruption for five or ten weeks, as characterized by lower progressive motility (PR) (16.19% vs. 28.63%, 5 weeks; 2.955% vs. 16.94%, 10 weeks), lower nonprogressive motility (NP) (28.82% vs. 29.7%, 5 weeks; 3.925% vs. 23.74%, 10 weeks) and higher IM (immotility) (54.99% vs. 41.67%, 5 weeks; 93.12% vs. 59.31%, 10 weeks) in the SC group than in the NC group (Fig. 3a-c). GNP treatment increased sperm motility after circadian disruption. Regarding the motion parameters of mouse sperm, the VAP variable did not differ in the samples treated with/without circadian disruption; however, the other two sperm kinetic values (VCL and VSL) decreased after five weeks of circadian disruption and genipin treatment restored it (Fig. 3d-f). Furthermore, circadian disruption and genipin treatment did not change BCF of sperm (Fig. 3g).

**Table 3:** Sperm concentration

	NC (Control)	SC (Disrupted + saline)	SG (Disrupted + GNP)
Sperm Con., ×10^6^	100.04 ± 35.53	33.22 ± 11.54^a^	102.55 ± 37.68^b^

### Changes in serum hormone levels

**Fig. 4:**
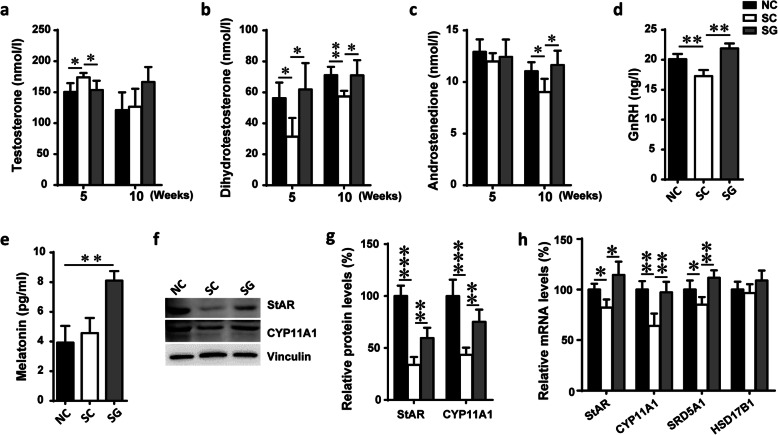
Hormones levels and the protein levels of StAR and CYP11A1 in the control and treated mice. **a** testosterone, **b** DHT, and **c** ASD in serum of control and treated mice for five or ten weeks. **d** Serum GnRH and (**e**) melatonin in control and treated mice for ten weeks. **f** The protein levels of StAR and CYP11A1. **g** The statistical quantification of panel **d**. **h** The mRNA levels of *StAR*, *CYP11A1*, *SRD5A1* and *HSD17B1* in the mouse testes under different treatments. **P < 0.01; ***P < 0.001

Hormone imbalance correlates with male infertility [37]. We collected the blood of NC, SC, and SG mice to evaluate the effect of circadian disruption, and genipin on hormone balance *in vivo*. We assessed serum testosterone, DHT, ASD, GnRH, and melatonin levels by ELISA. Circadian disruption for five weeks slightly increased testosterone levels (Fig. 4a). However, circadian disruption for five or ten weeks decreased the DHT, while ten weeks of circadian disruption decreased another male hormone, ASD (Fig. 4b and c). GNP treatment improved the serum levels of both of these hormones, which were diminished by circadian disruption. Moreover, the serum GnRH levels attenuated in mice after circadian disruption and were restored by GNP treatment (Fig. 4d). The melatonin levels showed no changes after circadian disruption and got upregulated by GNP treatment (Fig. 4e). As steroidogenesis is primarily mediated by the steroidogenic acute regulatory protein (StAR) and cytochrome p450 family 11 subfamily a member 1 (CPY11A1) [18], we measured the mRNA and protein levels of StAR and CYP11A1 in the testes of different treated mice. Western blot analysis indicated that the protein expression of StAR and CYP11A1 was significantly inhibited after circadian disruption, whereas genipin at least partially repaired the protein levels of StAR in testes (Fig. 4f and g). The mRNA levels of *StAR* and *CYP11A1* in mouse testes also decreased after the circadian disruption and upregulated by GNP treatment (Fig. 4h). Furthermore, we assessed the mRNA levels of *SRD5A1* and *HSD17B1*. qPCR results showed that GNP treatment augmented the mRNA levels of *SRD5A1*, which downregulated by circadian disruption, while the expression of HSD17B1 in mouse testes showed no significant change under the experimental conditions (Fig. 4h).

### Genipin regulates steroidogenesis in mouse Leydig tumor MLTC-1 cells

**Fig. 5:**
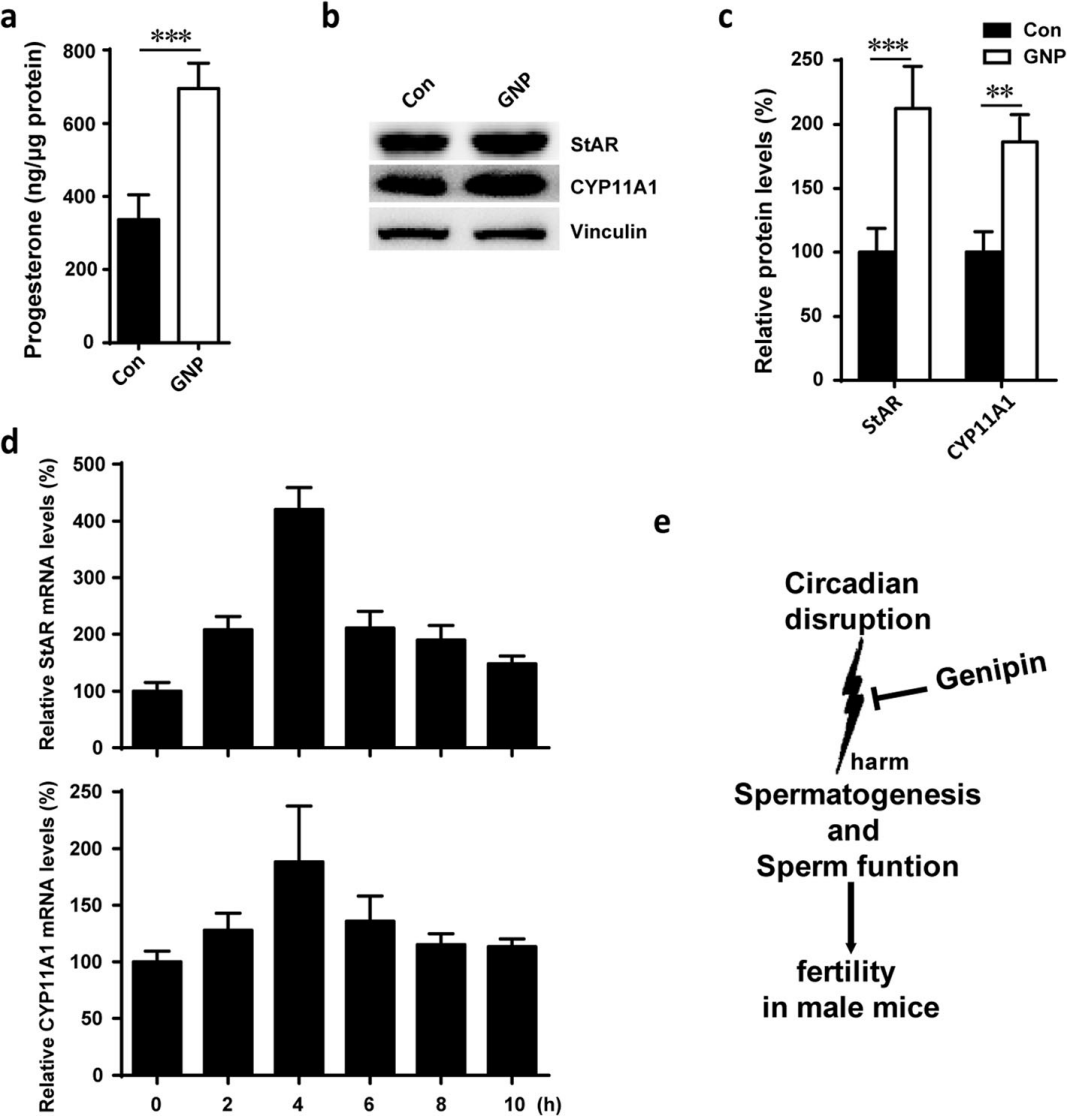
Genipin upregulates steroidogenesis in mouse ledig tumor MLTC-1 cells. **a** GNP increased progesterone secreted in MLTC-1. **b** Protein levels of StAR and CYP11A1 were upregulated by GNP in MLTC-1. **c** The statistical quantification of panel **b**. **d** mRNA levels of *StAR* and *CYP11A1*,in MLTC-1 after treated with or without GNP. **P < 0.01; ***P < 0.001. **e** The model of circadian disruption of spermatogenesis and fertility in male mice and its improvement by genipin

To further investigate the effect of genipin on steroidogenesis, MLTC-1 cells were cultured and treated with GNP. Following the addition of GNP for 24 hours, samples of the media were collected and analyzed for progesterone content by ELISA. The results in Fig. 5a demonstrate that treatment with GNP significantly increased the levels of progesterone secreted by MLTC cells. Next, we assessed the effects of GNP treatment on the mRNA and protein levels of StAR and CYP11A1 in MLTC cells. The western blot analysis results demonstrated that GNP treatments promoted StAR and CYP11A1 protein expression in MLTC-1 cells (Fig. 5b and c). The mRNA expression of both *StAR* and *CYP11A1* got upregulated by GNP after 2 to 4 hours of treatment (Fig. 5d). These results indicated that GNP increases steroidogenesis in MLTC-1 cells.

## Discussion

The Circadian rhythms are present in most living organisms from, eubacteria to humans, that most cells and tissues express autonomous clocks [1]. Disruption of circadian rhythms results in several kinds of metabolic disorders, revealing the interactions between metabolism and circadian rhythms at neural, molecular, and cellular levels [1, 2]. Circadian disruption can impact upon reproductive capacity, which is highlighted most obviously in mouse models with deletion or mutation of clock genes, resulting not only in circadian disruption, but also compromised male and female reproductive capacity [8]. Shift work gets associates with an increased risk of reproduction, such as endometriosis, infertility, miscarriage, and low birth weight in humans [21]. In this study, we identified that circadian disruption impairs spermatogenesis and fertility in male mice. Constant exposure to shortened T cycles increases the food intake and body weight gain of the mice. In male mice, it results in smaller litter size and lowers the average body weight of pups. Comparison with control conditions, circadian disruption significantly decreased sperm amounts. Similarly, the motility of sperm diminished. Genipin treatment remarkably enhances fertility and sperm parameters. Collectively, this evidence indicated that circadian disruption is detrimental to male fertility, and genipin treatment can improve this effect (Fig. 5e).

Reduced sperm concentration and diminished sperm motility can cause subfertility and infertility in mice [7]. In our study, circadian disruption reduced the sperm concentration by nearly 30% after ten weeks of exposure. Several factors may decrease sperm production, such as spermatogonia loss, cell cycle arrest, and intermediate stage sperm death. Morphological analysis revealed atrophy of the seminiferous tubules and a reduction in the number of spermatogonia and spermatids (Fig. 2). Although male infertility is multi-factorial, sperm motility is one of the most significant factors that regulate male fertility. In the SC mice, the decrease in sperm motility was time-dependent. Total motility decreased from 45–6.88% when the exposure time to shortened T cycles prolonged from five weeks to ten weeks. Although sperm motility reduced to less than 10%, the fertilization rate was approximately 50% in SC mice compared to 95% in NC mice. Our results were consistent with studies on human couples; male partners with less than 32% sperm motility were observed to be subfertile [38]. We observed high levels of sperm IM in the NC mice (> 40%), due to the exposure of the sperm extracted from the caudal epididymis to the experimental environment. These data are supported by reports that room temperature exposure causes a significant reduction in sperm motility and viability [39]. In addition to environmental factors, many genetic and physiological factors get implicated in sperm motility. At the molecular level, several signaling pathways and proteins are responsible for sperm motility [40]. However, the molecular mechanisms by which circadian disruption impairs spermatogenic function needs future elucidation. In addition to sperm motility, circadian disruption diminished two other sperm velocity parameters, VCL and VSL, while the average path velocity of sperm showed no changes among the three groups. All these results indicated that circadian disruption impaired spermatogenic function.

In male reproduction and spermatogenesis, sex hormones such as testosterone, DHT and ASD are the pivotal endocrine factors that control testicular functions and sexual behaviors [15, 37]. Steroidogenesis is a complex multistep and multienzyme process that is regulated by many factors, including tropic hormones and miRNAs [18, 41]. Previous studies showed that the dysregulation of circadian rhythm genes such as *Bmal1* and C*lock* results in the reduction of testosterone levels in serum [7, 14, 42]. The serum level of Testosterone displays circadian variation, and the change of testosterone level is dependent on sleep restriction rather than circadian disruption [43]. We detected a slight increase in testosterone levels in mice under circadian disruption in our study. These results may be due to the different L/D cycle between our control and circadian disruption group. Moreover, circadian disruption attenuated DHT, ASD, and GnRH levels in mice, while GNP treatment restored these hormones levels. The serum melatonin levels got strongly enhanced by GNP treatment. Furthermore, the protein levels of StAR and CYP11A1 decreased in the mouse testes after circadian rhythm disruption, indicating the transcriptional/posttranscriptional regulation of key steroidogenesis proteins by circadian rhythm.

Genipin is a natural extract from *G. jasminoides Ellis*, traditionally utilized as an herbal medicine in many Asian countries including, China. Also, as a natural crosslinker, GNP exerts antioxidant and anti-inflammatory effects by activating/inhibiting several signaling pathways [26, 28, 44]. On molecular levels,GNP was reported to regulate the expression of miR-142a-5p, which negatively regulated Srebp-1c, an important regulator of steroidogenesis and lipogenesis [27]. Furthermore, GNP shows the choleretic effect by enhancing bilirubin disposal and augment of genes in charge of the efflux of a number of organic anions [45]. GNP widely implicates in the inhibition of uncoupling protein 2 (UCP2) in various experimental models of ROS generation [45–47]. This study demonstrated the protective effect of GNP against circadian disruption-induced damage of spermatogenesis and fertility in male mice. Additionally, GNP treatment rescued the levels of the hormones DHT and ASD, as well as StAR, CYP11A1, and SRD5A1 mRNA and protein levels, in male mice under circadian disruption. In addition to the animal studies, GNP showed a positive effect by promoting steroid product secretion and the expression of key steroidogenic proteins (StAR and CYP11A1) in MLTC-1 cells. The protective effect of genipin may depend on its anti-oxidant and anti-inflammatory functions. Indeed, the circadian regulation of protein expression plays a significant role in the cellular response to oxidative stress and inflammation, whereas circadian disruption induced the augmentation of oxidative stress level and inflammatory response [48–50].

## Conclusions

In conclusion, this study shows that circadian disruption impairs spermatogenesis and fertility, which could be restored by genipin treatment in male mice. We showed that exposure to shortened T cycles reduces sperm concentration and motility in mice, resulting in decreased fertility index, litter size and pup body weight in mice. Furthermore, the levels of the serum reproductive hormones testosterone, DHT, ASD, and GnRH remained altered after exposure to shortened T cycles due to the dysregulation of StAR, CYP11A1 and SRD5A1 expression. Collectively, our findings may have implications for general health under circadian disruption and provide results that suggest genipin as a treatment.

## Data Availability

Not applicable.

## Abbreviations

DHT: Dihydrotestosterone
ASD: Androstenedione
StAR: Steroidogenic acute regulatory protein
CYP11A1: Cytochrome P450 family 11 subfamily A member 1
SRD5A1: Steroid 5α-reductase type 1
SCN: Suprachiasmatic nucleus
ADHD: Attention deficit hyperactivity disorder
NC: Normal control
ECD: Environmental circadian disruption
HPG: Hypothalamic-pituitary-gonadal
LH: Luteinizing hormone
FSH: Follicle-stimulating hormone
LD: Light-dark
GNP: Genipin
FBS: Fetal bovine serum
VAP: Average path velocity
VCL: Curvilinear velocity
VSL: Straight-line velocity
BCF: Beating cross frequency
STR: Straightness
LIN: Linearity
WOB: Wobble
HSD17B1: Hydroxysteroid 17-beta dehydrogenase 1
OMM: Outer mitochondrial membrane
IMM: Inner mitochondrial membrane
GnRH: Gonadotropin-releasing hormone
